# Meta-Analysis of Gene Expression Profiles in Acute Promyelocytic Leukemia Reveals Involved Pathways

**Published:** 2017-01-01

**Authors:** Mahdi Jalili, Ali Salehzadeh-Yazdi, Saeed Mohammadi, Marjan Yaghmaie, Ardeshir Ghavamzadeh, Kamran Alimoghaddam

**Affiliations:** 1 Hematology-Oncology and Stem Cell Transplantation Research Center, Tehran University of Medical Sciences, Tehran, Iran; 2 Department of Systems Biology and Bioinformatics, University of Rostock, 18051 Rostock, Germany

**Keywords:** Acute promyelocytic leukemia, Gene expression profile, Meta-analysis, Functional analysis

## Abstract

**Background:** Acute promyelocytic leukemia (APL) is a unique subtype of acute leukemia. APL is a curable disease; however, drug resistance, early mortality, disease relapse and treatment-related complications remain challenges in APL patient management. One issue underlying these challenges is that the molecular mechanisms of the disease are not sufficiently understood.

**Materials and Methods:** In this study, we performed a meta-analysis of gene expression profiles derived from microarray experiments and explored the background of disease by functional and pathway analysis.

**Results:** Our analysis revealed a gene signature with 406 genes that are up or down-regulated in APL. The pathway analysis determined that MAPK pathway and its involved elements such as JUN gene and AP-1 play important roles in APL pathogenesis along with insulin-like growth factor–binding protein-7.

**Conclusion:** The results of this meta-analysis could be useful for developing more effective therapy strategies and new targets for diagnosis and drugs.

## Introduction

 Acute promyelocytic leukemia (APL), classified as M3 in French-American-British (FAB) subtype classification system, is a bone marrow malignancy involving an excess of immature cells called promyelocytes. The cause of APL is a translocation between chromosomes 15 and 17, which consistently leads to breakage of the retinoic acid receptor-alpha (RARα) gene on chromosome 17. APL has unique clinical features, different responses to chemotherapy agents and a different molecular biology than other acute myeloid leukemias (AML). The incidence of APL accounts for 5–8% of all AML patients. APL is a treatable disease and currently around 90% of newly diagnosed patients achieve complete remission.^   1 ^ In addition, trials and clinical efforts are continuing to improve treatment results.^   2 ^ There are few treatment options for APL, including all-transretinoic acid (ATRA), as a single-agent therapy or combined with arsenic trioxide and/or other conventional chemotherapy drugs. The main challenges in treatment of APL currently include early mortality and relapse, refractory after induction of therapy^   3 ^ and drug resistance to ATRA and Arsenic trioxide (ATO).^   1 ^ The genetic and molecular aspects of APL are investigated more often than other human cancers^   4 ^ but try to increase knowledge of APL at molecular level is a key challenge that could lead to more effective treatment options. One area of focus is common fusion of RARα, which occurs in more than 98% of patients,^        5 ^ but there are six alternative fusion genes with different chromosomal translocations, which have been observed in rare cases and often lead to resistance to the most common therapies. ^6^^,^^7^  Based on the research cited above, the molecular and genetic mechanisms involved in APL pathogenesis and drug responses remain largely unknown. Detailed genomic analyses of functional and signaling pathways using clinical samples harvested from patients with APL may help with predicting prognosis, selecting effective targeting drugs, understanding molecular disease etiology and designing sophisticated new therapeutic strategies.

In recent years, biological studies have focused on holistic approaches such as using high-throughput and integrated multi-omics data and employing related tools such as graph theory and network analysis for biological investigations. ^8^^,^^9^  The integration of multi-omics data is a promising approach, which could resolve complexities in human biological systems as expected in systems biology.^   10 ^

There are two main methods for integration of omics data. In horizontal integration, the same data type such as multiple microarray gene profiles are combined, while in vertical integration data from different types such as microarray gene profiles and protein–protein interaction (PPI) are integrated. In first approach, the power of study is increased, particularly if the sample in each study is small. This method in microarray field is known as a meta-analysis. The meta-analysis can facilitate more reliable and valid results while decreasing individual and study-specific biases.^   11 ^

In this study, we performed a meta-analysis of available microarray gene profiles of human APL and normal samples and carried out functional analysis to create a list of differentially expressed genes (DEGs) as a biomarker signature for APL to determine functional features of this disease. Previous studies that used a reductionist approach have provided heterogenic results, whereas this study adopts a systematic and holistic approach. The results of this study may lead to novel pathways and/or drug targets in diagnosis and treatment of APL.

## MATERIALS AND METHODS

 Ramasamy et al.^   12 ^ developed a step by step approach for meta-analysis of microarray datasets. The outline of our study, according to this stepwise approach, is summarized in Table 1 in S2 Tables.

**APL Microarray Datasets**

The inclusion criteria comprised any human studies with at least two newly diagnosed APL patients and two corresponding normal human samples. Any surveys of cell lines, studies with patients who had other PML-RARA translocations, chromosomal aberrations except t (15;17) or treatments with any chemotherapy agents were excluded. For increased homogeneity of samples, only samples derived from bone marrow were selected because in accordance with the findings by Cheung et al.^13^ myeloblasts derived from bone marrow or from peripheral blood are different.

**Data Preprocessing and Quality Assessment**

The Affymetrix datasets raw data were loaded using Affy Bioconductor R package^   14 ^ and probe expression levels were extracted after quantile normalization and log base 2 scale transformation by rma function. For Illumina datasets, expression levels were obtained by get GEO function of GEOquery Bioconductor R package^   15 ^ and quantile normalization and log 2 transformation were also performed. Quality assessment for each dataset was performed to increase comparability and statistical power. In this study, we used array Quality Metrics,^   16 ^ a Bioconductor R package.

Because actual probe sequence information of chips was unavailable, it was not possible to ensure that the matched probes on the different platforms quantified the same mRNA transcript.^   17 ^ However, before cross-platform comparison, the probe sets must be mapped across various platforms to identify subsets of common genes. One probe-matching strategy is based on gene identifiers such as Unigene ID or Entrez Gene ID. Using Entrez Gene ID (previously Locus Link) may be a better approach compared Unigene ID because Entrez can map more genes between different platforms.^   18 ^ In this study, due to reasons mentioned, we used the Entrez Gene ID to match probes between different microarrays.

**Meta-analysis**

There are several methods for meta-analysis that have been described and compared in comprehensive review papers. ^19^^-^^21^  The technique selection for a meta-analysis depends on the objectives of the study and types of responses. There are four main methods for combining microarray information including vote counting, combining ranks, combining p-values, and combining effect sizes. More details about these methods and their variants have been described in the cited reviews and in valuable guidelines developed by Ramasamy et al.^   12 ^ Most statistics used for deferentially expressed gene analysis have been derived from pure mathematics and without biological consideration or certainty among biologists. However, rank product, which is a non-parametric statistical test, is more closely associated with biological reasoning. In fact, the rank product has helped discover genes that are consistently highly ranked in a genetic list.^   22 ^ As Hong et al.^   23 ^ discussed, this statistic has some advantages over others such as having more biological reasoning, fewer assumptions, high tolerance with noisy data and high performance when the number of replicates are low. The most important and applicable advantage of ranking is an increase in the results of sensitivity and reliability, especially when the data are heterogeneous.

The data in our study come from different platforms and different generations of the same platforms. The present study, therefore, uses the rank product statistic for its cross-platform, cross-generation meta-analysis.

**Functional and Gene Set Analysis**

Functional enrichment analysis is usually performed for the interpretation of genome-scale data via biologically relevant enriched labels in a gene list and comparisons to the corresponding distribution of other labels, using the rest of the genes as the background.^   24 ^

In this study, we performed several functional analyses for better interpretation of DEGs list, which was derived from meta-analysis. Gene ontology (GO)^   25 ^ is a commonly accepted and widely used method for functional studies, which organizes structured biological information for molecular function, biological processes and cellular components. The Reactome pathway database^        26 ^ is a manually curated and peer-reviewed human pathway and reactions resource. In this study, discovery of enriched functional-related gene groups (up-regulated and down-regulated genes) was performed using the Database for Annotation, Visualization and Integrated Discovery (DAVID)^   24 ^ tool, which is an integrated biological knowledge base and analytic tool.

For gene set enrichment analysis, GSEA Preranked^        27 ^ was used. It identified statistically significant, a priori–defined sets of genes by enriched sets and found correlations with the user-supplied ranking gene list. All genes that were used in the meta-analysis were rated according to a rank product analysis and used to create a ranked gene list. The Java GSEA Desktop Application version 2.2.1 was used and the enrichment statistic parameter set to basic but other parameters remained at their default values. Enrichment analysis was performed and scores were calculated based on the all gene set databases version 5.0 (c2.all.v5.0.symbols.gmt [curated]). Gene sets were considered significantly enriched at FDR q-values<0.01. For identifying up-stream regulators among significantly altered genes derived from meta-analysis, data were analyzed through use of QIAGEN’s Ingenuity Pathway Analysis (IPA, QIAGEN, Redwood City, CA, USA). The default setting was used and z-score was used for inferring a significant activation state (z-score>2) or inhibition state (z-score<−2).

**PPI Network Construction and Analysis**

Proteins control all internal and external activities of a cell and interactions among these proteins, which were studied via PPI networks, playing important roles for biological interpretation.^28^

STRING^        29 ^ version 10 was used for the construction and analysis of the PPI network using up and down-regulated genes obtained from the meta-analysis as input. Only interactions with experimental and knowledge evidences with high confidence scores (0.7) were selected. The constructed network was visualized and topologically analyzed using Cytoscape version 3.2.1 and Network Analyzer Plugin^   30 ^ version 2.7.

For more evaluation of deregulated genes, a network enrichment analysis (SNOW)^   31 ^ was done. The SNOW program extracts a sub-network from the interactome that was prepared from different public PPI repositories using a given set of genes. SNOW first maps all given genes onto the interactome and calculates a minimum connected network (MCN) defined as the shortest network that connects all interacting nodes within a given gene list. SNOW calculates several relevant network parameters and conducts corresponding tests to assess their significance for the interactome and topological parameters against a set of created MCNs by random proteins. The filtered scaffold interactome, proteins whose interactions were detected by at least two different experimental methods, was selected and allowed to add external intermediate nodes which are not present in our list. For revealing the related functional features of MCN members, we used EnrichNET,^   32 ^ a network-based enrichment analysis tool, to calculate association scores between our MCN proteins and protein sets of Reactome database pathways.

## Results

 After applying inclusion criteria, four datasets from 4 different studies were collected and analyzed. These datasets included 63 untreated and newly diagnosed APL patient samples, t (15;17) (q22;q12) without any additional cytogenetic abnormalities and 28 normal human controls. Detailed dataset information is shown in Table 1. The quality assessment of all datasets was performed using array Quality Metrics Bioconductor package and described in the S1 Methods in detail.

**Table 1:** Datasets includes in the meta-analysis (See also S1 Methods)

**Data set**	**Patient**		**Normal**	
	**Reported**	**After QA** *	**Reported**	**After QA** *
GSE1159	21	18	5	4
GSE12662	11	10	10	4
GSE34823	25	22	8	8
GSE43176	6	3	5	2

* : Quality assessment

All APL or normal sample profiles that did not meet sufficient quality standards were marked as poor and removed from our final analysis. In total, 53 of 63 APL disease gene profiles and 18 of 28 normal samples were selected for analysis. The goal of the gene mapping step was to provide a probe or gene list that comprised all selected gene expression datasets. Because our datasets included different platforms, obtaining a common probe list was not possible due to differences in designs of platform probe sequences. Therefore, we created a list of common genes among the datasets according to their Entrez gene IDs. One-to-many and many-to-many gene mapping were done according to the methods described in the S1 Methods. As a result of this step, we had an Entrez gene ID list with 12710 rows, where Entrez gene IDs were mapped to official gene symbols using the Bioconductor annotation packages.

Meta-analysis was performed on 4 datasets with 12710 genes that were available on all datasets. The product rank statistics was used for meta-analysis and the analytical results identified 647 up-regulated genes and 465 down-regulated genes in APL patients compared to normal human controls. In order to increase robustness and reduce the number of deregulated genes, an additional leave-one-out analysis was performed as described in the S1 Methods. Top gene selection criteria consisted of FDR<0.01 and a fold change ratio ≥1.5. Finally, a gene signature of 247 up-regulated and 159 down-regulated genes was selected after the leave-one-out analysis for future functional analyses. Tables 2 and 3 display the top 20 up and down-regulated genes and the complete list displayed in Tables 2 and 3 in S2 Tables. As expected, a large number of genes were deregulated due to the systemic nature of APL. To further investigate the top deregulated genes, functional analyses including GO analysis, pathway analysis and pre-ranked gene set enrichment analysis were carried out. The results of the over-representation analysis performed using DAVID tools displayed in Table 4 in S2 Tables.

All 12710 genes involved in meta-analysis were ordered according to up or down-regulation. These genes were then used to create a pre-ranked, ordered gene list and a pre-ranked gene set enrichment analysis was performed by Broad GSEA Preranked using all pathways’ gene sets. The GSEA analysis resulted in a large number of gene sets that enriched positively and negatively in the ranked gene list. The top 10 gene sets are displayed in Table 4 and full list are found in S3 File. The Ingenuity Upstream Regulator Analysis was performed and significantly activated and inhibited regulators identified. The activated or inhibited regulators are displayed in Table 5 and in Table 5 in S2 Tables.

**Figure 1:**
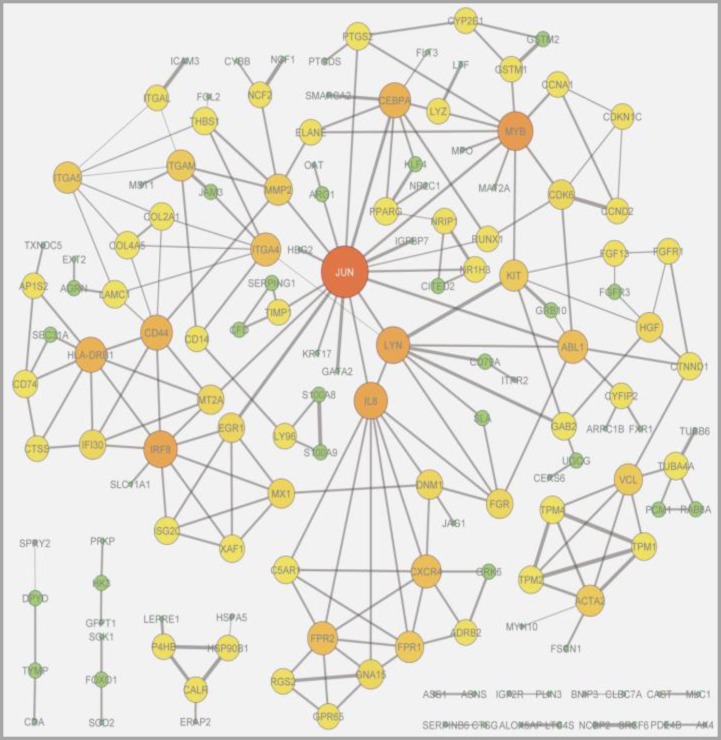
Protein-protein interaction network constructed by up and down-regulated genes derived from meta-analysis of APL gene expression profiles. All disconnected nodes were removed. Network analysis displayed a hub role for JUN (jun proto-oncogene) which was up-regulated. The size of nods indicates the degree of nodes and weight of edges indicate the combination of STRING experimental and knowledge evidence score. The network is enriched in interactions (p-value=1.75e-2)

To determine the biological network among selected up and down-regulated genes, all deregulated genes were mapped to the STRING PPI network by employing high confidence scores (0.7) on experiments and databases as active prediction methods (Figure 1). The mapped network contained 217 interactions among 146 proteins. All other proteins showed no interactions and were removed from the network.

**Table 2:** List of top 20 up-regulated genes (FDR<0.01 and Fold change >1.5)

**No**	**Entrez ** **gene ID**	**Gene ** **symbol**	**Gene name**
**1**	1359	CPA3	carboxypeptidase A3 (mast cell)
**2**	8900	CCNA1	cyclin A1
**3**	23166	STAB1	stabilin 1
**4**	3485	IGFBP2	insulin-like growth factor binding protein 2, 36kDa
**5**	3082	HGF	hepatocyte growth factor (hepapoietin A; scatter factor)
**6**	1675	CFD	complement factor D (adipsin)
**7**	54360	CYTL1	cytokine-like 1
**8**	7177	TPSAB1	tryptase alpha/beta 1
**9**	481	ATP1B1	ATPase, Na+/K+ transporting, beta 1 polypeptide
**10**	710	SERPING1	serpin peptidase inhibitor, clade G (C1 inhibitor), member 1
**11**	6320	CLEC11A	C-type lectin domain family 11, member A
**12**	6624	FSCN1	fascin actin-bundling protein 1
**13**	445	ASS1	argininosuccinate synthase 1
**14**	10765	KDM5B	lysine (K)-specific demethylase 5B
**15**	5954	RCN1	reticulocalbin 1, EF-hand calcium binding domain
**16**	10225	CD96	CD96 molecule
**17**	2322	FLT3	fms-related tyrosine kinase 3
**18**	9452	ITM2A	integral membrane protein 2A
**19**	1287	COL4A5	collagen, type IV, alpha 5
**20**	2769	GNA15	guanine nucleotide binding protein (G protein), alpha 15 (Gq class)

A topological analysis of the protein interaction network revealed a diameter of 9 and network density of 0.021. Analysis displayed a hub role for JUN (jun proto-oncogene) with 17 interactions. The JUN gene was up-regulated. This gene is a transcription factor that binds to the enhancer heptamer motif 5′-TGA [CG] TCA-3′ and also activates the NR5A1 when phosphorylated by HIPK3. This cascade increases steroidogenic gene expression via cAMP signaling pathway. Other high-degree nodes included MYB (v-myb avian myeloblastosis viral oncogene homolog, up-regulated), CEBPA (CCAAT/enhancer binding protein (C/EBP), alpha, up-regulated), CXCL8 or IL8 (interleukin-8, down-regulated), LYN (LYN proto-oncogene, Src family tyrosine kinase, down-regulated) and IRF8 (interferon regulatory factor 8, down-regulated).

**Table 3:** List of top 20 down-regulated genes (FDR<0.01 and Fold change> 1.5)

**NO**	**Entrez ** **gene ID**	**Gene ** **symbol**	**Gene name**
**1**	6283	S100A12	S100 calcium binding protein A12
**2**	50486	G0S2	G0/G1 switch 2
**3**	79887	PLBD1	phospholipase B domain containing 1
**4**	728	C5AR1	complement component 5a receptor 1
**5**	6280	S100A9	S100 calcium binding protein A9
**6**	929	CD14	CD14 molecule
**7**	2357	FPR1	formyl peptide receptor 1
**8**	10288	LILRB2	leukocyte immunoglobulin-like receptor, subfamily B (with TM and ITIM domains), member 2
**9**	2219	FCN1	ficolin (collagen/fibrinogen domain containing) 1
**10**	25797	QPCT	glutaminyl-peptide cyclotransferase
**11**	6279	S100A8	S100 calcium binding protein A8
**12**	11031	RAB31	RAB31, member RAS oncogene family
**13**	115207	KCTD12	potassium channel tetramerization domain containing 12
**14**	2268	FGR	FGR proto-oncogene, Src family tyrosine kinase
**15**	50856	CLEC4A	C-type lectin domain family 4, member A
**16**	8870	IER3	immediate early response 3
**17**	3101	HK3	hexokinase 3 (white cell)
**18**	4082	MARCKS	myristoylated alanine-rich protein kinase C substrate
**19**	653361	NCF1	neutrophil cytosolic factor 1
**20**	1520	CTSS	cathepsin S

**Table 4:** Result of gene set enrichment analysis (GSEA Preranked)

**Top of the ranked gene sets name (FDR q-val<0.01)**	**SIZE**	**NES**	**FDR q-val**
ROSS-AML-WITH-PML-RARA-FUSION	70	7.968	0.000
JAATINEN-HEMATOPOIETIC-STEM-CELL-UP	218	5.853	0.000
CASORELLI-ACUTE-PROMYELOCYTIC-LEUKEMIA-UP	159	5.550	0.000
MULLIGHAN-MLL-SIGNATURE-2-DN	269	5.394	0.000
IVANOVA-HEMATOPOIESIS-EARLY-PROGENITOR	365	5.265	0.000
CAIRO-HEPATOBLASTOMA-UP	203	5.027	0.000
VERHAAK-AML-WITH-NPM1-MUTATED-DN	237	4.962	0.000
PENG-GLUTAMINE-DEPRIVATION-DN	327	4.920	0.000
SHEN-SMARCA2-TARGETS-UP	415	4.764	0.000
VALK-AML-CLUSTER-12	29	4.741	0.000
**Bottom of the ranked gene sets name (FDR q-val<0.01)**	**SIZE**	**NES**	**FDR q-val**
JAATINEN-HEMATOPOIETIC-STEM-CELL-DN	196	-11.905	0.000
MCLACHLAN-DENTAL-CARIES-UP	235	-8.740	0.000
GOLDRATH-ANTIGEN-RESPONSE	302	-8.515	0.000
MULLIGHAN-MLL-SIGNATURE-2-UP	397	-8.440	0.000
VERHAAK-AML-WITH-NPM1-MUTATED-UP	179	-7.729	0.000
POOLA-INVASIVE-BREAST-CANCER-UP	268	-7.695	0.000
MCLACHLAN-DENTAL-CARIES-DN	226	-7.656	0.000
MULLIGHAN-MLL-SIGNATURE-1-UP	361	-7.588	0.000
SMID-BREAST-CANCER-NORMAL-LIKE-UP	451	-7.512	0.000
ROSTY-CERVICAL-CANCER-PROLIFERATION-CLUSTER	137	-7.486	0.000

**Table 5:** Ingenuity Upstream Analysis result

**Up-stream** **Regulator**	**Molecule Type**	**Predicted** **Activation** **State**	**Activation** **z-score**
**MAPK1**	kinase	Activated	2.625
**IRF4**	transcription regulator	Activated	2.621
**MAPK9**	kinase	Activated	2.219
**TREM1**	transmembrane receptor	Activated	2.019
**TGM2**	enzyme	Inhibited	-5.386
**IFNG**	cytokine	Inhibited	-3.229
**NFkB (complex)**	complex	Inhibited	-3.142
**TNF**	cytokine	Inhibited	-2.743

For increased robustness of PPI network construction and analysis, If PPI network analysis is SNOW network enrichment, this is correct. All deregulated genes were used along with an MCN obtained by Babelomics 5 suite. The MCN comprised 368 proteins, which included 192 (52.2%) genes that were differentially expressed, and 176 (47.8%) genes from external nodes. The MCN was compared with random networks obtained from given genes with the same size, more connections (connectivity degree p-value=0.05), higher connectivity (clustering coefficient p-value= 0.01) and more hub nodes (betweenness centrality p-value=0.05). To detect any possible associations between the MCN proteins and cellular pathways, we used EnrichNet,^   32 ^ a network-based protein set enrichment analysis tool. The Reactome pathways were selected for pathway-representing reference gene sets and used a modified STRING PPI network by Bossi and Lehner for PPI network, which solely included experimentally verified and direct physical interactions. Gene set network similarity ranking (gene set vs. pathways) was performed and pathways which had XD-scores greater than the significance threshold (1.03) are displayed in Table 6 in S2 Tables.

## Discussion

 APL is one of a few diseases that have an effective drug for targeted therapy and it has been studied in more depth than other acute leukemia subtypes. In addition to its unique genetic profile, several biological and molecular features contribute to establishment of APL as a distinct unit within the acute myeloid leukemias. Some of these characteristics are relevant because of their impact on disease clinical presentation and use of targeted treatment but the roles of others in pathogenesis and responses to therapy are more controversial. Large clinical trials such as AML17 study^2^ have continued to achieve higher survival rates, reduced side effects and prevention of early mortality and disease recurrence.

**Figure 2:**
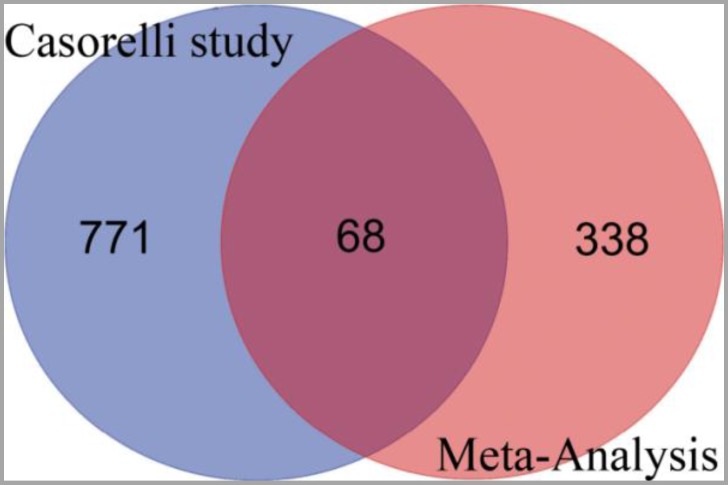
Venn diagram of deregulated genes of Casorelli et al.33 study and our meta-analysis which showed 68 common genes among two studies. Casorelli et al. study curated genes list obtained from Molecular Signatures Database (MSigDB) version 5.0. The common genes include ANKS1A, CD44, STT3A, DHCR24, PFKP, CCNA1, COL4A5, ELOVL5, AP1S2, ATP1B1, SERPING1, ALCAM, QPRT, CTSW, NRIP1, IGFBP7, PRDX4, JAG1, SPRY2, GFI1, SKAP2, SLA, HLA-DPA1, SCRN1, MYH10, FDFT1, GABRE, TPM1, ACOT9, TRIB1, TIMP1, RGS2, IVNS1ABP, CITED2, PTGDS, DMXL2, BLVRA, FLT3, STAB1, MXRA7, SERPINB6, SAP30, HTATIP2, THBS1, ALDH1A1, SLC16A3, KRT18, ID2, XBP1, NRIP3, ZNF185, VCL, CD96, FGF13, HLA-DRB1, SLC38A1, FLOT1, GATA2, N4BP2L1, VNN2, JUN, AUTS2, HDC, AAK1, HGF, CFD, CKAP4 and PDE3B.

Few studies have investigated APL using high-throughput data with human samples. Casorelli et al.^   33 ^ obtained eight denovo APL patient samples, eight secondary APL samples and compared them to eiaght normal CD34+ samples. According to their report, 1020 genes were differentially expressed. The authors focused on DNA repair genes and showed that inefficient base excision repair and recombinational repair have roles in APL molecular processes. The results of the present meta-analysis and Casorelli et al. showed 68 overlapping genes (Figure 2).

We performed a meta-analysis of high-throughput gene expression data of APL patients followed by a set of functional analyses. As was expected in DEG 

analysis, a vast number of deregulated genes were found to be involved in APL and extensive alterations in various processes of APL were also revealed. The PPI network analysis of altered genes confirmed that some genes play significant roles in this network (Figure 1) and among them, JUN (jun proto-oncogene) gene has a hub role in the PPI network. Mitogen-activated protein kinase (MAPK1 and MAPK9) regulators were activated with high scores according to the Upstream Regulator Analysis prediction program. In addition, pathway analysis showed that the activator protein-1 (AP-1) family of transcription factors was activated in our study. The AP-1 family belongs to the class of basic leucine zipper (bZIP) transcription factors, which are necessary for dimerization and DNA binding. It binds to promoters of its target genes in a sequence-specific manner and transactivates or suppresses them. The Jun (c-Jun, Jun-B and Jun-D) and Fos (c-Fos, FosB, Fra1 and Fra2) subfamilies are the major AP-1 proteins. The AP-1 proteins are involved in the regulation of a variety of cellular processes, including proliferation and survival, differentiation, growth, apoptosis, cell migration, and transformation.^34^ The regulation of AP-1 activity is critical for cell fate and occurs at various levels including through dimer-composition, transcriptional and posttranslational events and through interaction with accessory proteins. Frequently, increased AP-1 levels lead to enhanced transactivation of target gene expression.^35^^,^^36^ C-Jun is at the center stage of molecular network with mysterious functional properties and is the most broadly studied protein of AP-1 complex. Recent research has divulged multiple layers of a complex regulatory scheme in which c-Jun is able to crosstalk, amplify and integrate different signals for tissue development and disease.^   37 ^ Jun-B transcriptionally regulates the expression of cyclin A and was remarkably the first AP-1 protein found to do so.^   38 ^

Molecular mechanisms have elucidated the ability of Jun-B to function as a cell cycle inhibitor and tumor suppressor gene via down-regulation of cyclin D expression and up-regulation of p16INK4A expression as a tumor suppressor inhibiting CDK4 and CDK6 genes.^39^^,^^40^ CDK6 was enriched in PPI network in the present study. Moreover, research using a knock-in strategy and a transgenic complementation approach has verified that Jun-B is required for cell cycle re-entry after quiescence.^41^

AP-1 activity can also be regulated by post-translational modification including phosphorylation by MAPKs. The MAPK family includes extracellular signal regulated kinase (ERK), p38 MAPK and c-Jun N-terminal kinase (JNK). The exact mechanisms of specific conditions and treatments on AP-1 activation and the relative roles of different MAPKs in these processes are diverse.^42^

AP-1 is known to be involved in TNF-α receptor signaling pathway, allowing TNF-α to influence the expression of many genes.^   43 ^ TNF-α is also important for development and progression of a number of types of cancer. The up-regulation of TNF-α is involved in cell growth and proliferation via NF-κB-dependent or -independent pathways in tumors. Positive feedback between NF-κB and TNF-α promotes leukemia-initiating cell capabilities.^   44 ^

The p38 MAPK signaling pathway is also activated by cellular stimuli that exert negative regulatory effects on hematopoiesis. P38 MAPK appears to be activated by myelo suppressive cytokines such as TGF-β and TNF-α.^45^^,^^46^ Additionally, p38 MAPKs also perform posttranscriptional regulation of cytokines such as TNF-α and IL-1. Therefore, p38 MAPK signaling has been implicated in processes ranging from apoptosis to cell cycles, induction of expression of cytokine genes and differentiation.^47^^-^^50^

All forms of MAPK cascades have been found to take part in the regulation of AP-1. Recently, MAPK signaling has been demonstrated to play a key role in the maintenance of hematopoietic stem cell (HSC) quiescence.^   51 ^ Examination of normal HSCs has shown that a significant fraction of stem cells were quiescent because they remained in the G0 phase of the cell cycle, whereas ROS-mediated activation of p38 resulted in abolition of quiescence in HSCs. The connection between oxidants and stem cell aging has been further supported by a study reporting that ROS-related oxidative stress abrogates the reconstructing capacity of HSCs, leading to defective self-renewal of HSCs.^   52 ^ Jang et al. showed that the ROS^low^ HSC population has a higher self-renewal potential, whereas significant HSC exhaustion was observed in the ROS^high^ population following serial transplantation, which agrees with our findings in APL. The p38 MAPK activity was higher in the ROS^high^ compared to the ROS^low^ population.^   52 ^

Alsayed et al.^   53 ^ reported that the p38 MAPK pathway plays a negative role in the induction of ATRA responses in APL and raises the possibility that combined use of ATRA with pharmacological inhibitors of p38 might be more effective than the use ATRA alone. Similarly, treatment of NB-4 acute promyelocytic leukemia cells with arsenic trioxide resulted in the activation of the p38 MAPK, which activated the protein kinase 2 pathway, whereas pharmacological inhibition of p38 further enhanced arsenic trioxide-induced apoptosis and growth inhibition of APL which have not been well specified.^   54 ^

Our findings were comparable to the results of Geh et al.^55^ with regard to the MAPK cascade in APL. They observed that the enzyme activities of MAP3K1 were required to activate the JNK-c-Jun cascade. Thus, MAP3K1 and c-Jun form an intracrine regulatory loop in which c-Jun controls MAP3K1 expression, while MAP3K1 in turn controls c-Jun N-terminal phosphorylation and AP-1 activity.

IGF-1R are membrane receptors and their ligand binding by the insulin-like growth factor-1 (IGF-1) leads to receptor phosphorylation and activation of MAPK and PI3K/Akt signaling.^   56 ^ In our study, the GAB1 signalosome pathway was significantly enriched (Table 6 in S2 Tables). GAB1 is engaged to the activated EGFR indirectly through GRB2. GAB1 acts as an adaptor protein that enables formation of an active PIK3 via recruitment of PIK3 regulatory subunit, which leads to the activation of the AKT signaling.

The activity of IGF-1R is closely controlled by its ligands. Ligand bioavailability is partly controlled by the family of secreted insulin-like growth factor-binding proteins (IGFBP1 to IGFBP6),^57^ (IGFB-related protein 1, also known as insulin-like growth factor-binding protein-7 (IGFBP7). In our meta-analysis, IGFBP2 and IGFBP7 were significantly up-regulated. It has been shown that high expression levels of this protein are accompanied by the growth of several types of tumors. In parallel with our observations, Verhagen et al.^   58 ^ reported that IGFBP7 sensitizes AML cells to chemotherapy-induced cell death. Moreover, overexpression of IGFBP7, as well as addition of recombinant human IGFBP7, is able to reduce survival of AML cells by the induction of a G2 cell cycle arrest and apoptosis. Importantly, in that study, 102 non-M3 AML patients with high IGFBP7 expression had better outcomes than patients with low IGFBP7 expression, indicating a positive role for IGFBP7 in the treatment and patient outcomes of AML. Taken together, this suggests that the combination of IGFBP7 and chemotherapy potentially overcomes conventional AML drug resistance and thus improves AML patient survival.

The CD86 (B7.2) molecules are surface glycoproteins and members of the Ig super family that are expressed only on professional antigen presenting cells (APCs). They are important in the early interactions between APCs and T cells during the induction of immune response. It is well established that mCD86 is expressed by AML myeloblasts in a considerable proportion of patients with acute myeloid leukemia in which substantial number of patients have expressed CD86 molecules.^59^^,^^60^ In our pathway analysis, CD86 stayed on downstream of inhibited regulators; so, it is expected that CD86 downregulated in APL patients. Hamed et al.^   61 ^ reported that sCD86 levels are highest in FAB subtypes with highest AML blast levels, which results in poor prognosis. Those findings strongly suggest that sCD86 is derived from the malignant cells in those patients.

Our meta-analysis clearly demonstrates down-regulation of RNA binding protein RBM38, which is involved in neutrophil differentiation in APL. These results are supported by the results from a recent study by Wampfler et al.^   62 ^ where the expression of the RNA binding proteins RBM38 and DND1 were repressed in primary AML patients, and neutrophil differentiation was dependent on increased expression of both proteins.

## CONCLUSION

 This study used a meta-analytical approach to develop a gene signature for APL containing 406 genes that are up or down-regulated. According to pathway analysis, the MAPK pathway and its involved elements such as the JUN gene and AP-1 play important roles in APL pathogenesis. IGFBP7 was shown to be altered and could be a target in APL. The results of this meta-analysis could be useful for future studies that could lead to the development of more effective therapeutic strategies and new targets for diagnostic procedures and drug development.
